# Commentary: A Host-Produced Quorum-Sensing Autoinducer Controls a Phage Lysis-Lysogeny Decision

**DOI:** 10.3389/fmicb.2019.01171

**Published:** 2019-06-03

**Authors:** Claudia Igler, Stephen T. Abedon

**Affiliations:** ^1^Institute of Science and Technology Austria, Klosterneuburg, Austria; ^2^Department of Microbiology, The Ohio State University, Mansfield, OH, United States

**Keywords:** communication, extracellular signaling, ecology, induction, prophage, temperate

With the recent publication by Silpe and Bassler (2019), considering phage detection of a bacterial quorum-sensing (QS) autoinducer, we now have as many as five examples of phage-associated intercellular communication (Table 1). Each potentially involves ecological inferences by phages as to concentrations of surrounding phage-infected or uninfected bacteria. While the utility of phage detection of bacterial QS molecules may at first glance appear to be straightforward, we suggest in this commentary that the underlying ecological explanation is unlikely to be simple.

**Table 1:** Phage-associated intercellular communication mechanisms.

**Mechanism^1^**	**Phage type**	**Signal**	**Receptor or reception mechanism**	**Lysis delayed or accelerated? (context of outcome)^2^**	**Selected by what densities of phage-infected bacteria?**	**Selected by what densities of phage-uninfected bacteria?**	**Summary**	**References^3^**
Lysis inhibition	T-even coliphages	Phage virions	Secondary adsorption	Delayed (inhibition of lysis during lytic cycles)	High^4^	Low^5^, ^6^	In the presence of high densities of free phages, lysis of lytic infections is delayed (inhibited), as stimulated by secondary phage adsorptions	Abedon, 1990, 1994, 2008, 2009; Ramanculov and Young, 2001; Tran et al., 2005, 2007; Moussa et al., 2012, 2014; Chen and Young, 2016
Synchronized lysis-inhibition collapse	T-even coliphages	Phage virions	Secondary adsorption	Accelerated (lysis from without of otherwise lysis-inhibited lytic cycles)	High^7^	Low	In the presence of high densities of free phages, lysis of lytic infections is accelerated, as stimulated by secondary phage adsorptions	Abedon, 1992, 1994, 1999, 2009
High-multiplicity lysogeny decision	Temperate phages	Phage virions	Secondary infection, coinfection, superinfection^8^	Delayed (bias toward initiation of lysogenic cycles at start of phage infections)	High	Low	In the presence of high densities of free phages, lysis of infections is delayed (as lysogenic cycles), as stimulated by phage coinfection	Avlund et al., 2009; Joh and Weitz, 2011
Arbitrium system	Temperate phage Phi3T of *Bacillus subtilis*	Phage-produced hexapeptide molecules	Phage-encoded receptor	Delayed (bias toward initiation of lysogenic cycles at start of phage infections)	High	Low	In the presence of high densities of phage-infected bacteria, lysis of phage infections is delayed (as lysogenic rather than as lytic cycles), as stimulated by presence of phage-encoded autoinducer molecules	Erez et al., 2017; see also Hargreaves et al. (2014) for phage-encoding of a possible autoinducer system
Host autoinducer	Temperate Phage VP882 of *Vibrio cholerae*	Host-produced DPO molecules	Phage-encoded receptor	Accelerated (prophage induction terminating lysogenic cycles)	Low^9^	High^10^	In the presence of high densities of bacteria, lysis of infections is accelerated (via prophage induction), as stimulated by presence of bacteria-encoded autoinducer molecules	Silpe and Bassler, 2019; see also Ghosh et al. (2009) and references cited therein for potentially ecologically analogous systems, as well as Hargreaves et al. (2014) who posit prophage induction as a possible utility to phage-encoded autoinducer systems

1 *Mechanisms are shown in approximate order of discovery (except synchronized lysis-inhibition collapse, which was discovered third)*.

2 *Acceleration of lysis can represent induction of a new lytic cycle or instead earlier-than-expected-lysis during an existing lytic cycle*.

3 *See Abedon (2017a) for pre-1990, less ecologically-oriented references for the first three mechanisms*.

4 *In each case, relatively high densities of phage-infected bacteria are problematic due to their potential to inactivate adsorbing, clonally related virions such as via superinfection exclusion or superinfection immunity*.

5 *Relatively high densities of phage-uninfected bacteria would result in lower likelihoods of secondary adsorption or secondary infection, thereby reducing the expression of the various so-dependent phenotypes, such as lysis inhibition, etc*.

6 *As applicable to this and the next three rows, lysis delay aspects of phenotypes can give rise to increased virion production per phage-infected bacterium – or, in the case of temperate phages, vertical replication of lysogens ultimately leading to increased numbers of induction events – both of which are phage-desirable mechanistic responses to low densities of uninfected bacteria, e.g., Abedon et al. (2003, 2009)*.

7 *It is the lysis of these high densities of phage-infected bacteria that is accelerated by a synchronization of lysis-inhibition collapse*.

8 *These terms are being used effectively synonymously*.

9 *Given receptor expression under these conditions (as so far has not been explicitly demonstrated), then this system could be induced by high densities of phage-infected bacteria (lysogens supplying autoinducer) but such lysogenic bacteria, if present at high densities, could adsorb, and inactivate clonally related free phages due to superinfection immunity; hence, high densities of phage-infected bacteria should select against autoinducer-based phage-induction mechanisms rather than for such mechanisms*.

10 *Though it is likely that accelerated lysis would be selected by the presence of high densities of phage-uninfected bacteria, just how high densities of phage-uninfected bacteria would be supplied to released virions is uncertain, i.e., as considered in the main text of this commentary, particularly the first scenario: Phage-susceptible targets*.

## Autoinducer-Motivated Prophage Induction

In a fascinating study, Silpe and Bassler (2019) show that a temperate vibriophage can both detect and respond to a host-produced QS autoinducer, 3,5-dimethylpyrazin-2-ol (DPO). This cue helps to determine the phage's lifestyle via a mechanism that is potentially ecologically similar to observations provided by Ghosh et al. (2009). DPO normally represses *Vibrio cholerae* biofilm formation, resulting in biofilm dispersion (Papenfort et al., 2017). Silpe and Bassler (2019) found that VP882, a linear-plasmid prophage, encodes a host-homologous DPO receptor, VqmA_Phage_, which responds to extracellular DPO by stimulating production of Qtip, an antirepressor of the lysogenic repressor, CI. Resulting CI sequestration activates the phage's lytic pathway and thereby production and release of new VP882 virions. Qtip production also leads to induction of closely related vibriophages that encode non-homologous antirepressor analogs. What remains unclear, as indicated by the authors, is under which circumstances the phage QS receptor is expressed and also, our emphasis here, *under what circumstances might DPO detection be beneficial to a phage*.

## Ecological Scenarios

Bacterial quorums represent high densities of bacteria (Waters and Bassler, 2005), as could be beneficial to phages undergoing lytic cycles (first scenario, below), though not necessarily always (Saucedo-Mora et al., 2017). DPO-motivated prophage induction, however, might offer more subtle benefits, and below we consider six scenarios of varying phage utility. Local bacterial populations are assumed to consist at least initially of individual, clonal microcolonies (Costerton et al., 1985; Kreft, 2004; Nadell and Bassler, 2011; van Gestel et al., 2015).

### Phage-Susceptible Targets

VP882 lysogens might be found among non-VP882 infected, phage-susceptible, DPO-supplying bacteria. Here prophage induction presumably would be directly beneficial to the phage. From Silpe and Bassler (2019), “This strategy likely maximizes phage infection of the next *V. cholerae* cell.” For example, DPO could bias VP882 vibriophages *newly infecting* a *V. cholerae* microcolony toward sooner lytic cycles, or a spatially distinct VP882 lysogen microcolony might be situated in close association with a VP882-uninfected *V. cholerae* microcolony.

### Phage-Resistant Targets

VP882 lysogen microcolonies might be found among bacteria which are *not* VP882 susceptible, e.g., as due to any of various bacterial phage-resistance strategies (Hyman and Abedon, 2010; Labrie et al., 2010). Induction of a lytic cycle in this case would not be of obvious local benefit to the phage. Indeed, induction could be particularly harmful to VP882 if local, resistant bacteria were still adsorbable, but not infectible.

### Sister-Lysogen Targets

Due to superinfection immunity (Blasdel and Abedon, 2017), clonally related, fellow lysogens would be adsorbable but not infectable (Ghosh et al., 2009). Unless prophage presence were to dampen DPO production, then resulting induction among sister lysogens could result in virion release into environments consisting of high densities of virion-inactivating bacteria. This scenario is perhaps more plausible than Scenario 1, given the founding of microcolonies by single lysogens, ones not necessarily surrounded by phage-sensitive *V. cholerae* strains. Prophage induction could still result in virion dissemination to new cells that are found at more distant locations, though with the utility of this dissemination being *despite* dangers of released virion exposure to virion-inactivating, neighboring lysogens. The following three scenarios build on this scenario.

### Danger and Opportunity of Dispersal

Upon dispersal, i.e., as is associated with DPO detection, biofilm bacteria can be especially metabolically active and additionally, by breaking free of microcolonies, potentially more vulnerable to lytic phages (Sauer et al., 2002; Abedon, 2017b; Vidakovic et al., 2018). DPO thereby could in part serve as a “danger” signal to a prophage, one analogous to detection of DNA damage (Ptashne, 2004), but signaling instead increased lysogen vulnerability to unrelated lytic phages. At the same time, improved bacterial metabolism at the point of biofilm dispersal could be capable of supporting particularly vigorous induced VP882 lytic cycles. Indeed, depending on the timing of VP882 induction, resulting lysis might not occur until following lysogen dispersal away from the danger of adsorbable fellow lysogens.

### Bet Hedging (<<100% Induction)

Induction despite sister lysogens, if limited in extent, could be viewed as a bet-hedging strategy (Avlund et al., 2009; Maslov and Sneppen, 2015; Xue and Leibler, 2018). DPO exposure, perhaps given varying DPO or VqmA_Phage_-expression levels across microcolonies, thereby could give rise to phage populations existing as robust combinations of both virions and lysogens, each possessing distinct dispersal strategies and vulnerabilities. Thus, VP882 virions are *less* vulnerable to unrelated phages, but more vulnerable to VP882 lysogens. VP882 lysogens instead are *more* vulnerable to unrelated phages, invulnerable to fellow VP882 lysogens, and better able (via longer infections) to take advantage of host motility to disperse to new locations.

### Near 100% Induction

If lysogens are locally exposed to equivalent levels of DPO, then this might trigger *en masse* lysis of sister lysogens, reducing their potential to inactivate released virions (similarly, see synchronized lysis-inhibition collapse, Table 1). This too could be bet-hedging, should somewhat smaller numbers of uninduced lysogens remain with reduced vulnerability to unrelated phages, having entered a so-called “numerical refuge” (Chao et al., 1977).

## Conclusion

Better understanding DPO-motivated prophage induction will require determining the *in situ* timing of VqmA_Phage_-receptor expression and its ecological context, e.g., such as the spatial distribution of its or DPO's presence within phage VP882 lysogen microcolonies. Regardless of specifics, integrating host-derived signals presumably increases the environmental information obtained by a prophage. Perhaps, then, the timing and context of *VqmA*_*Phage*_ expression can predispose DPO-motivated prophage induction toward circumstances which are more favorable to virion release than might be seen within dense populations of sister lysogens.

## Author Contributions

All authors listed have made a substantial, direct and intellectual contribution to the work, and approved it for publication.

### Conflict of Interest Statement

The authors declare that the research was conducted in the absence of any commercial or financial relationships that could be construed as a potential conflict of interest.
